# Mouse Apolipoprotein B Editing Complex 3 (APOBEC3) Is Expressed in Germ Cells and Interacts with Dead-End (DND1)

**DOI:** 10.1371/journal.pone.0002315

**Published:** 2008-05-28

**Authors:** Chitralekha Bhattacharya, Sita Aggarwal, Madhu Kumar, Amatul Ali, Angabin Matin

**Affiliations:** Department of Cancer Genetics, University of Texas, MD Anderson Cancer Center, Houston, Texas, United States of America; Victor Chang Cardiac Research Institute, Australia

## Abstract

**Background:**

The *dead-end* (*Dnd1*) gene is essential for maintaining the viability of germ cells. Inactivation of *Dnd1* results in sterility and testicular tumors. The *Dnd1* encoded protein, DND1, is able to bind to the 3′-untranslated region (UTR) of messenger RNAs (mRNAs) to displace micro-RNA (miRNA) interaction with mRNA. Thus, one function of DND1 is to prevent miRNA mediated repression of mRNA. We report that DND1 interacts specifically with APOBEC3. APOBEC3 is a multi-functional protein. It inhibits retroviral replication. In addition, recent studies show that APOBEC3 interacts with cellular RNA-binding proteins and to mRNA to inhibit miRNA-mediated repression of mRNA.

**Methodology/Principal Findings:**

Here we show that DND1 specifically interacts with another cellular protein, APOBEC3. We present our data which shows that DND1 co-immunoprecipitates APOBEC3 from mammalian cells and also endogenous APOBEC3 from mouse gonads. Whether the two proteins interact directly remains to be elucidated. We show that both DND1 and APOBEC3 are expressed in germ cells and in the early gonads of mouse embryo. Expression of fluorescently-tagged DND1 and APOBEC3 indicate they localize to the cytoplasm and when DND1 and APOBEC3 are expressed together in cells, they sequester near peri-nuclear sites.

**Conclusions/Significance:**

The 3′-UTR of mRNAs generally encode multiple miRNA binding sites as well as binding sites for a variety of RNA binding proteins. In light of our findings of DND1-APOBEC3 interaction and taking into consideration reports that DND1 and APOBEC3 bind to mRNA to inhibit miRNA mediated repression, our studies implicate a possible role of DND1-APOBEC3 interaction in modulating miRNA-mediated mRNA repression. The interaction of DND1 and APOBEC3 could be one mechanism for maintaining viability of germ cells and for preventing germ cell tumor development.

## Introduction

The *Dnd1* gene is essential for primordial germ cell survival in vertebrates from zebrafish, xenopus to mice [1]–[3]. Loss of *Dnd1* expression in primordial germ cells results in their death during embryogenesis, thus causing sterility in adults. In 129 strain mice, inactivation of *Dnd1*, as in the 129-*Ter* mouse strain, causes loss of germ cells and in addition, development of testicular germ cell tumors [3]. Germ cell tumor development in 129-*Ter/Ter (Dnd1-/-*) mice is also initiated during embryonic stages [4]–[7].

Based on analysis using the NCBI conserved domain database (CDD), the mouse *Dnd1* protein, DND1, has at least one RNA recognition motif (RRM) [3]. DND1 has been shown to bind to the 3′-UTR of mRNAs to displace miRNAs that bind to adjacent sites on the same mRNA [8]. miRNA are small RNAs (20–22 nt) that regulate gene expression, mainly by post-transcriptional processes [9]–[11]. Evidence indicates that binding of DND1 to the 3′-UTR of p27 or LATS2 mRNA hinders access of miR-221 or miR-372, respectively [8], [12]. This results in increased p27 or LATS2 protein levels because DND1 inhibits miRNA-mediated repression of protein expression.

As DND1 is specifically expressed in germ cells, it serves to differentially regulate expression of mRNA in germ cells. During early development, germ cells are set aside from the somatic cells of the embryo on the basis of differential regulation of mRNA in germ cells compared to somatic cells [13], [14]. For example, in zebrafish germ cells, *Dnd1* binds to *nanos1* and *tdrd7* mRNA to displace miR-430 binding to mRNA. This prevents miR-430 mediated inhibition of *nanos1* and *tdrd7* expression in germ cells [8]. In contrast, miR-430 inhibits *nanos1* and *tdrd7* expression in somatic cells because somatic cells do not express *Dnd1*. These observations provide an explanation as to why lack of *Dnd1* in germ cells causes loss of expression of germ cell specific proteins therefore resulting in germ cell death. However, it is not clear why lack of *Dnd1* causes germs cells to become transformed as in the *Ter* mouse strain. One possibility is that absence of DND1 may cause deficiency of tumor suppressor proteins and this may cause germ cell tumor development.

**Figure 1:**
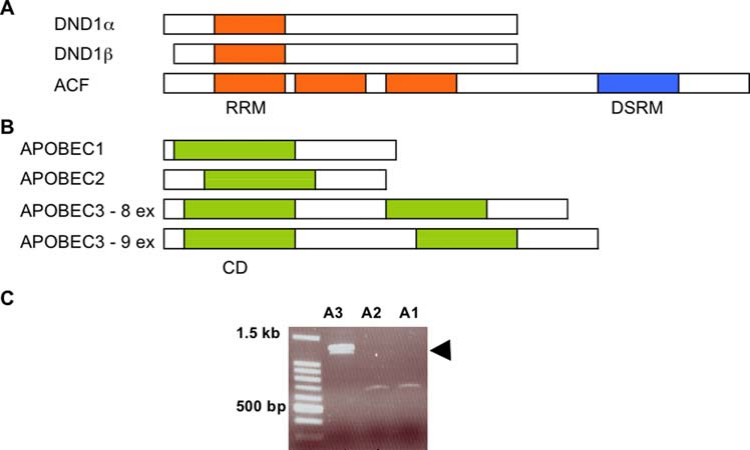
(a) Comparing DND1 and ACF. Representation of DND1α, DND1β and ACF proteins, drawn to scale. RNA recognition motif (RRM) is indicated in orange. ACF possesses 2 additional RRMs not found in DND1 as well as a double-stranded RNA binding motif (DSRM). The protein structure analyses are from NCBI conserved domain database (CDD). (b) Comparing APOBEC proteins. The protein coding regions of APOBEC1, APOBEC2, APOBEC3 (8 exon isoform) and APOBEC3 (9 exon isoform) are compared. The cytidine deaminase (CD) domains are indicated in green. APOBEC3 possesses 2 cytidine deaminase domains, which are not functionally equivalent. The protein structure analyses are from NCBI conserved domain database (CDD). (c) RT-PCR amplifies *Apobec1*, *Apobec2* and *Apobec3* from mRNA of 129 mouse testes. Transcripts of both isoforms of *Apobec*3 (with 8 and 9 exons, respectively) are indicated by the doublet band (arrow).

Mouse DND1 protein shows highest homology to ACF (APOBEC1 complementation factor) [5], [3]. The overall amino acid identity between DND1 and ACF is 34%. There is 48% identity between the RNA recognition motif (RRM) of DND1 and the first RRM of ACF (Fig. 1a). ACF is the essential RNA-binding co-factor of APOBEC1 and together they comprise the RNA editing enzyme complex (editosome) [15], [16]. APOBEC1 together with ACF converts specific cytidines to uridines in the apolipoprotein B transcript and other mRNAs. On the basis of the homology between DND1 and ACF, we hypothesized that DND1, like ACF, may interact with APOBEC-like proteins.

APOBEC proteins are present in vertebrates from zebrafish to man [17]. Members of the family have zinc-binding domains with homology to bacterial cytidine deaminase. The APOBEC family members in the mouse that have RNA or DNA deaminase activity, or both are: APOBEC1, APOBEC2, APOBEC3 and AICD (activation-induced cytidine deaminase; also known as AID). The function of APOBEC2 is not known. It is expressed mainly in muscle cells [18] and has lower, but still detectable cytidine deaminase activity compared to APOBEC1. APOBEC3, is expressed in the testes, adult germ cells [19] and a wide variety of tissues. Human APOBEC3 is able to hypermutate the cDNA derived during replication of exogenous and endogenous retroviruses in cells. Thus APOBEC3 inhibits viral replication [20] and functions to maintain innate immunity of cells. AICD is found mainly in B-cells and is involved in class-switch recombination and somatic hypermutation of antibody genes [21]. Deamination within the immunoglobulin loci by AICD initiates antibody gene diversification. A new *Apobec* gene family member, *Apobec4* was recently identified by genomic analysis but its protein and enzyme function has not been determined [22]. Mice and humans encode one gene each for APOBEC1, APOBEC2, APOBEC4 and AICD. The mouse genome also encodes one *Apobec3* gene on chromosome 15. However, in humans there has been an expansion of the *APOBEC3* gene family such that eight *APOBEC3* genes are tandemly encoded on human chromosome 22q13.1 [17], [19]. These genes are designated *APOBEC3A* to *APOBEC3H*. Of these, APOBEC3G and APOBEC3F have been extensively studied because of their ability to restrict human immunodeficiency virus (HIV) [23]–[25].

Aberrant expression of APOBECs in the cell has been proposed to cause promiscuous nucleotide modification and genomic instability [26]. Human APOBEC1 and APOBEC3 exhibit DNA mutator activity in *Escherichia coli* assays [27]. They caused DNA mutation through cytidine (dC) deamination and demonstrated target sequence specificity. Thus, APOBEC proteins are potential mutators and this has possible implications in cancer development. Because APOBECs have the potential ability to mutate cellular nucleic acids, their activity in cells is likely to be tightly regulated.

Recent studies on APOBEC3 indicate that it possesses cellular functions in addition to its ability to restrict retroviruses and retrotransposons. Human APOBEC3 was found to prevent microRNA-mediated inhibition of protein translation [28]. This did not require the cytidine deaminase activity of APOBEC3G. In addition, a number of reports now show that APOBEC3G interacts with a variety of RNA-binding proteins and locates to P-bodies and stress granules [29]–[31]. Very interestingly, this novel function of APOBEC3 parallels the role of DND1 in inhibiting miRNA function.

We investigated whether mouse DND1 can interact with any of the APOBEC proteins. Here, we report that DND1 can interact specifically with APOBEC3. In light of recent reports pertaining to modulation of miRNA function by both DND1 and APOBEC3, it is likely that these two proteins may act synergistically or oppose each other to modulate miRNA function.

## Results

### Isolation of Apobec cDNAs from testes of 129 mice

We previously found that inactivation of *Dnd1* results in sterility as well as increased testicular tumors in the 129 mouse strain [3]. The DND1 protein shows highest overall homology to ACF (APOBEC1 complementation factor) (Fig. 1a). We sought to determine if DND1, like ACF [15], [16], has the ability to interact with any of the APOBEC family proteins that are normally expressed in mouse testes.

As a first step, we determined the *Apobec* transcripts that are present in mouse testes. We designed primers to specifically amplify *Apobec* transcripts from RNA derived from the testes of the 129 mouse strain. RT-PCR amplified *Apobec1*, *Apobec2* and two isoforms of *Apobec3* (Fig. 1c). Sequencing of the RT-PCR products indicated that we had isolated two isoforms of *Apobec3* that differ because they contain 8 and 9 exons, respectively (Fig. 1b). The 8-exon *Apobec3* isoform has an amino acid sequence identical to that of the 9-exon isoform except that it lacks 33 amino acids in the central part of the protein. The 8-exon isoform is translated from an alternately spliced mRNA that skips exon 5 of *Apobec3* yet retains the same reading frame. These two isoforms of mouse *Apobec3* have been previously described in the database [http://www.ensembl.org/Mus_musculus/geneview?gene=ENSMUSG00000009585].

Although *Apobec1* and *Apobec2* have not previously been reported to be present in the testes, we were able to detect their transcripts by RT-PCR indicating that low levels of these *Apobec*s are in fact present in the testes. We were unable to isolate the *AICD* transcript from testes RNA possibly because its expression is restricted to B-lymphocytes. The full-length cDNAs of the *Apobec* genes were cloned into pBK-CMV vectors (Stratagene), sequence-verified and used as templates for *in vitro* transcription and translation reactions to generate [^35^S]methionine-labeled APOBEC proteins.

### DND1 interacts with APOBEC proteins in vitro

**Figure 2:**
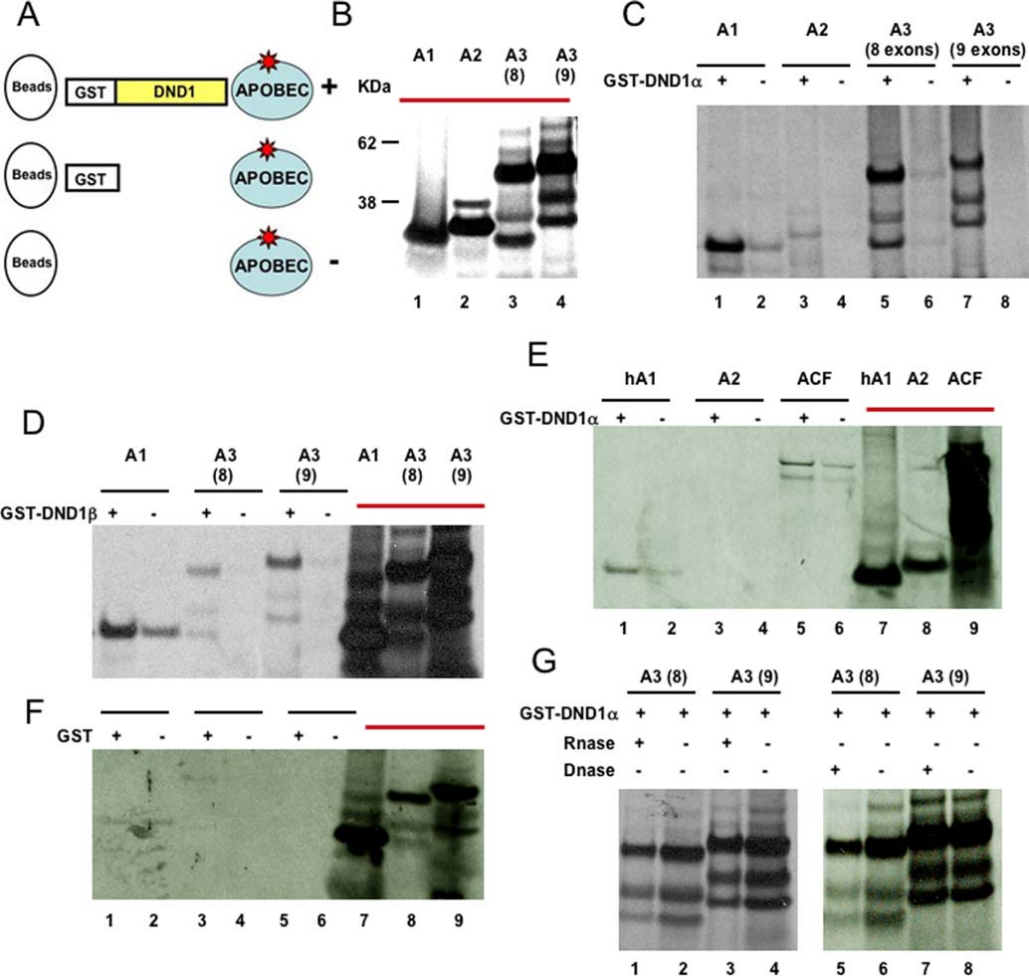
DND1 interacts with APOBEC3 *in vitro.* (a) Experimental design to test interaction of DND1 to the APOBEC proteins *in vitro*. All reactions contained [^35^S]methionine-labeled APOBEC proteins (blue circles). The labeled proteins were incubated with purified GST-DND1 (indicated by+in panels c–e). Glutathione Sepharose 4B beads (beads, white circles) were added to the reactions. The beads are able to bind to the GST moiety of DND1 and should also “pull-down” proteins that associate with GST-DND1. Control incubations used beads alone incubated with labeled APOBEC proteins (indicated by–in panels c–e). (b) *In vitro* transcribed/translated [^35^S]methionine-labeled APOBEC proteins. 5 μL was loaded from each translation reaction. A1 (APOBEC1), A2 (APOBEC2), A3 (8) (8-exon isoform APOBEC3) and A3 (9) (9-exon isoform of APOBEC3). The translated proteins used as input are marked with red bars (in panels b, d–f). (c) Binding of APOBEC proteins to GST-DND1α. 40 μL of [^35^S]methionine-labeled APOBEC proteins was incubated with GST beads and either with (+) or without (−) GST-DND1α. Lanes 5 and 7 show binding of APOBEC3 to GST-DND1α. Lanes 6 and 8 show no binding of APOBEC3 to the beads. (d) Binding of APOBEC proteins to GST-DND1β. 40 μL of [^35^S]methionine-labeled APOBEC proteins (APOBEC1 and both isoforms of APOBEC3) were incubated with GST beads and either with (+) or without (−) GST-DND1β. Lanes 3 and 5 show binding of APOBEC3 to GST-DND1β. Lanes 4 and 6 show no binding of APOBEC3 to the beads. Lanes, 7, 8 and 9 are the translated proteins used as input (5 μL). (e) Testing interaction of human APOBEC1 and ACF to GST-DND1α. 40 μL of [^35^S]methionine-labeled human APOBEC1 (hA1), mouse APOBEC2 (A2) and human ACF were incubated with GST beads and either with (+) or without (−) GST-DND1α. Comparison of lanes 1 and 2 indicate weak binding of human APOBEC1 (hA1) to GST-DND1α. Comparison of lanes 5 and 6 indicate no specific binding of ACF to GST-DND1α. Lanes, 7, 8 and 9 are the translated proteins used as input (5 μL). (f) Binding of APOBEC proteins to GST (second control). 40 μL of [^35^S]methionine-labeled APOBEC proteins (APOBEC1 and both isoforms of APOBEC3) were incubated with GST beads and either with (+) or without (−) GST. Lanes, 7, 8 and 9 are the translated proteins used as input (5 μL). (g) Effect of RNase (left) and DNase (right) on APOBEC3 and GST-DND1 interaction. [^35^S]methionine-labeled APOBEC proteins incubated together with GST-DND1α̃ and GST beads were divided into two. Each aliquot was treated with RNase (+) or DNAse (+) or not treated (−) prior to electrophoresis.

The TnT Coupled Reticulocyte Lysate Systems (Promega) was used to generate [^35^S]methionine-labeled APOBEC proteins in *in vitro* transcription and coupled translation reactions (see Experimental Procedures section) (Fig. 2b). For controls, human APOBEC1 (hA1) and human ACF were also generated by *in vitro* transcription/translation of cloned expression constructs (Fig. 2e). The sizes and amount of translated [^35^S]methionine-labeled APOBEC protein products were determined by electrophoreses.

To test whether any of the APOBEC family members can interact with DND1 *in vitro*, [^35^S]methionine-labeled APOBEC proteins were incubated with purified GST(glutathione S-transferase)-DND1 [32] (Fig. 2a). Glutathione (GST) Sepharose 4B beads were then added to the mix. In control reactions, equal amounts of [^35^S]methionine-labeled APOBEC proteins were incubated with beads only. The glutathione Sepharose 4B beads bind to the GST moiety of GST-fusion proteins (in this case, GST-DND1) and should therefore “pull-down” proteins that associate with GST-DND1. At the end of the incubation period, the Sepharose 4B beads were pelleted and washed to reduce non-specific binding. The proteins bound to the beads were released into loading dye by heating, prior to electrophoreses. In a second control, [^35^S]methionine-labeled APOBEC proteins were incubated with purified GST protein and beads (Fig. 2a and f).

These results show that APOBEC3 is able to specifically bind to GST-DND1. Both isoforms of APOBEC3, the 9-exon isoform and the 8-exon isoform, showed specific binding to GST-DND1α (Fig. 2c, lanes 5–8) and GST-DND1β (Fig. 2d, lanes 3–6). The binding of APOBEC3 is specific to the DND1 moiety of GST-DND1 because APOBEC3 did not bind to GST protein (Fig. 2f) or to the beads (Fig. 2c, lanes 6 & 8, and Fig. 2d, lanes 4 & 6). Mouse and human APOBEC1 were also able to bind to GST-DND1 but there was significant non-specific binding in the control lanes (Fig. 2c, d and e, lanes 1 & 2). Thus, APOBEC1 may also be able to bind to DND1 but with lower affinity compared to APOBEC3. APOBEC2 or ACF did not bind to GST-DND1 (Fig. 2e).

Further, we tested whether the *in vitro* binding of GST-DND1 to APOBEC3 is dependent on RNA or DNA that may be present in the *in vitro* transcription/translation reactions. However, treatment of [^35^S]methionine-labeled APOBEC3-GST-DND1 complexes with excess RNase or DNase did not affect binding of the two proteins (Fig. 2g). This indicates that any RNA or DNA present in the TnT coupled transcription/translation rabbit reticulocyte lysates (Promega) was not involved in promoting binding of APOBEC3 to DND1. In summary, the data indicates that APOBEC3 is able to specifically bind to DND1. These experiments provided the first evidence that DND1 can interact directly with APOBEC3.

### DND1 interacts with APOBEC3 in mammalian cells

**Figure 3:**
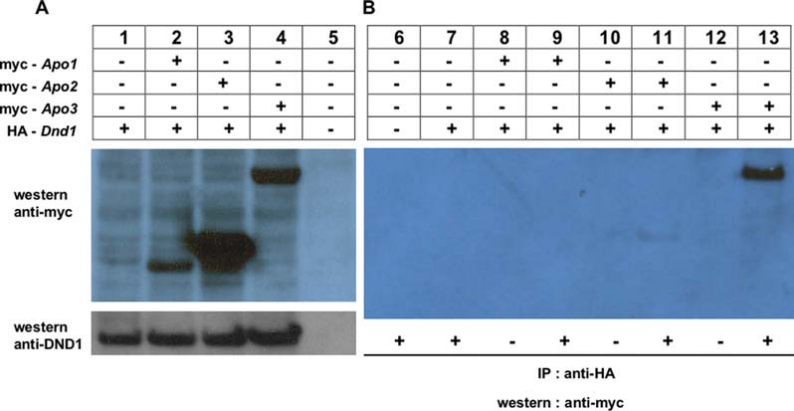
DND1 interacts with APOBEC3 in mammalian cells. (a) Expression of APOBEC proteins and DND1 in 293T transfected cells. Cells were co-transfected with expression vectors encoding HA-tagged *Dnd1* (lanes 1, 2, 3 & 4) and myc-tagged *Apobec1, Apobec2* and *Apobec3* (lanes 2, 3 & 4, respectively). After 48 h, transfected cells were lysed. An aliquot (10 μg) was electrophoresed for western blotting with anti-myc (top panel) and anti-DND1 antibody (bottom panel) to detect expression of proteins. (b) HA-DND1 pulls-down APOBEC3 from cells. Lysates from transfected cells in (a) were used. Equal aliquots (50 μg) were used for immunoprecipitation (IP) using anti-HA antibody (+) to pull down HA-DND1 (lanes 6, 7, 9, 11 & 13). Control lanes had no antibody (−). Following IP, electrophoresis and transfer to membranes, western blotting was performed using anti-myc antibody.

Next, we tested whether DND1 can interact with APOBEC proteins in mammalian cells. HA-tagged *Dnd1* and myc-tagged *Apobec* plasmid constructs were co-transfected into 293T cells. myc-APOBEC1, 2, and 3 expression in transfected 293T cells were detected by immunoblotting with anti-myc (Fig. 3a, top panel). Expression of HA-tagged DND1 was also detected using anti-DND1 antibody, antibody C [32] (Fig. 3a, lower panel). The lysates were used for immunoprecipitation with anti-HA antibody to “pull-down” HA-DND1 and associated proteins. No antibody was added to controls. After immunoprecipitation with anti-HA antibody, electrophoresis and transfer to membranes, western blotting was performed using anti-myc antibody.

The results showed that myc-APOBEC3 was “pulled-down” specifically with HA-DND1 (Fig. 3b, lane 13). In contrast, myc-APOBEC1 and myc-APOBEC2 were not “pulled-down” by HA-DND1 (Fig. 3b, lanes 9 and 11).

These results are evidence that DND1 can associate with APOBEC3 in mammalian cells. This *in vivo* pull-down of tagged proteins is a second technique used to validate the previous *in vitro* binding results. Interaction of APOBEC3 to GST-DND1 *in vitro* is likely because of direct binding of APOBEC3 to GST-DND1 whereas “pull-down” of myc-APOBEC3 by HA-DND1 from cell lysates suggests interaction of the proteins but which may not necessarily be due to direct binding. Additional co-factors present in mammalian cells could bridge the interactions between the HA-DND1 and myc-APOBEC3 proteins. To summarize, the *in vivo* pull down experiment in 293T cells demonstrates that DND1 and APOBEC3 are present in the same protein complexes in mammalian cells.

### DND1 “pulls-down” endogenous APOBEC3 from mouse testes

We next tested whether DND1 interacts with APOBEC3 in mouse tissues. Both DND1 and APOBEC3 are expressed in adult testes [32], [33], [3]. Because antibodies for immunoprecipitation of DND1 are currently not available, we used purified GST-DND1 to perform *in vivo* “pull-down” experiments. We incubated GST-DND1 protein with mouse testes lysate. The GST-DND1 was then incubated with Sepharose 4B beads to pull down GST-DND1 and any associated proteins. The beads were subsequently washed, and then boiled in loading dye prior to electrophoresis and transfer to membranes. To detect APOBEC3, immunoblotting was performed with polyclonal, rabbit anti-mouse APOBEC3 antibody (Upstate). This antibody detects an epitope in the N-terminus of mouse APOBEC3.

**Figure 4:**
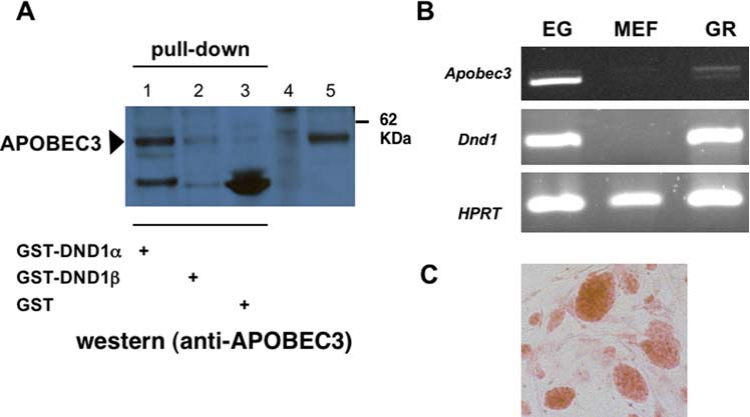
DND1 and APOBEC3 in mouse tissues. (a) DND1 interacts with endogenous APOBEC3. Mouse testis lysates were incubated with GST-DND1α (lane 1), GST-DND1β⪽ (lane 2) or GST protein (lane 3) and incubated with Sepharose 4B beads. The beads were washed and the contents eluted into loading dye prior to electrophoresis and immunoblotting with rabbit anti-mouse APOBEC3 antibody. Control lanes on the left are cell lysates from untransfected cells (lane 4) and lysates from cells transfected with myc-APOBEC3 expression constructs (lane 5). (b) *Dnd1 and Apobec3* are expressed in embryonic germ (EG) cells and genital ridges. RT-PCR for *Apobec3*, *Dnd1* and *HPRT* (hypoxanthine-guanine phosphoribosyltransferase) on total RNA from EG cells, mouse embryo fibroblasts (MEF), and E13.5 genital ridges (GR). (c) EG cells in culture, stained with alkaline phosphatase (orange clusters), growing on MEF cell layer.

Our results showed that GST-DND1α was able to “pull-down” APOBEC3 from mouse testes (Fig. 4a). APOBEC3 from mouse testis was the same size as myc-APOBEC3 expressed in cells transfected with *myc-Apobec3* expression vector (Fig. 4a). In this experiment, GST-DND1β was not able to efficiently “pull-down” APOBEC3 from mouse testes and this suggests that DND1α isoform interacts more efficiently with endogenous, testes APOBEC3. DND1α isoform is expressed in germ cells during embryogenesis as well as in adult stages. In contrast, DND1β is expressed in meiotic and post-meiotic germ cells in adult mice [32].

### Apobec3 transcripts in mouse germ cells and developing gonads

Germ cell tumors arise in the developing gonads (genital ridges) of the male embryo. *Dnd1* transcripts are detected in germ cells soon after germ cell specification [34] and in mouse embryonic gonads (genital ridges) [32], [3]. *Dnd1* encoded protein, DND1, is also expressed in embryonic germ (EG) cells grown in culture [32], [3]. EG cells are totipotent cells and have been isolated from primordial germ cells of embryos of embryonic (E) 8.5 –E 11.5 stages from hybrid strains of B6C3 F1 mice [35], [36]. Although expression of *Apobec3* in adult tissues has been described [37], the expression of *Apobec3* in embryonic stages has not been examined. We therefore examined the expression of both *Dnd1* and *Apobec3* transcripts in embryonic germ (EG) cells and in genital ridges (embryonic gonads).

RT-PCR indicated the presence of *Apobec3* and *Dnd1* transcripts in EG cells (Fig. 4b). *Apobec3* and *Dnd1* were not detected in RNA from mouse embryo fibroblasts (MEFs), which are the feeder cell layers on which EG cells grow (Fig. 4c).

In addition, we also isolated embryonic gonads (genital ridges) from 129 strain, male E13.5 embryos. RT-PCR for *Apobec3* and *Dnd1* transcripts was carried out on total RNA prepared from the E13.5 genital ridges. *Dnd1* and *Apobec3* transcripts are expressed in E13.5 genital ridges (Fig. 4b). Interestingly, RT-PCR indicated that EG cells express higher levels of the shorter, 8-exon isoform of *Apobec3* compared to that in the genital ridges. One reason for this could be because the EG cells are derived from a different mouse strain, B6C3 hybrid, whereas the genital ridges are from 129. In conclusion, we demonstrate that both *Apobec3* and *Dnd1* are expressed in embryonic gonads and in embryonic germ cells of the mouse.

### DND1 and APOBEC3 sequester to peri-nuclear sites

**Figure 5:**
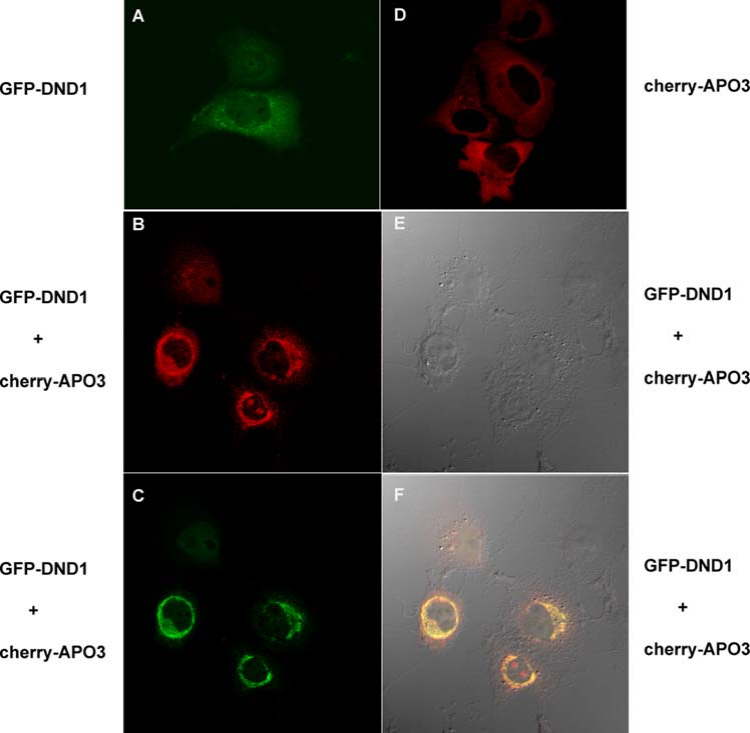
Mouse DND1 and APOBEC3 co-localize to peri-nuclear sites in the cell cytoplasm. (a) COS7 cells were transfected with expression constructs for GFP-*Dnd1* for visualization of green fluorescence due to GFP-DND1. Cells were fixed with 2% paraformaldehyde before visualization of fluorescent signal by confocal microscopy. (d) COS7 cells were transfected with expression constructs for mCherry-*Apobec3* for visualization of red fluorescence due to mCherry-*Apobec3* by confocal microscopy. (b,c,e and f) COS7 cells were co-transfected with expression constructs for GFP-DND1 and mCherry-*Apobec3*. (b) Red fluorescence due to mCherry-APOBEC3 was imaged using red filter (excitation at 548 nm). (c) Green fluorescence due to GFP-DND1 was imaged using green filter (excitation at 488 nm). (f) The merged image of cells co-transfected with GFP-*Dnd1* and mCherry-*Apobec3*, which shows GFP and mCherry signal co-localized at the peri-nuclear regions of the cells. (e) The DIC (differential interference contrast) image of the cells.

To visualize the cellular localization of the DND1 and APOBEC3, we transfected expression plasmids encoding GFP-*Dnd1* and mCherry-*Apobec3* into different mammalian cells such as COS7, NIH3T3 and 293T cells. The transfected cells were observed using a *Zeiss* LSM 510 confocal microscope. Overall, when either GFP-DND1 or mCherry-APOBEC3 were transfected in cells, the expression of the fluorescent-tagged proteins was predominantly in the cytoplasm (Fig. 5a and d). However, when both GFP-DND1 and mCherry-APOBEC3 were co-transfected in COS7 cells, we observed a sharp overlap of the green and red signals to form a ring-like zone surrounding the nucleus indicating co-localization of the GFP-DND1 and mCherry-APOBEC3 fluorescent signals to perinuclear sites (Fig. 5b, c and f). The co-localization suggests that DND1 and APOBEC3 likely sequester each other.

**Figure 6:**
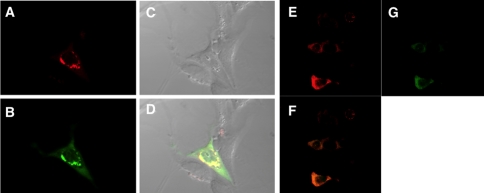
Localization of APOBEC3 and DND1 in NIH3T3 and 293T cells. (a–d) Co-transfection of mCherry-Apobec3 and GFP-DND1 expression constructs into NIH3T3 cells. Cells were fixed with 2% paraformaldehyde before visualization of fluorescent signal by confocal microscopy. (a) Red fluorescence due to mCherry-APOBEC3 was imaged using red filter (excitation at 548 nm); (b) green fluorescence due to GFP-DND1 was imaged using green filter (excitation at 488 nm); (d) merged image of red and green filters showing APOBEC3 and DND1 co-localize (yellow signal) near perinuclear region in NIH3T3 cells. (c) The DIC (differential interference contrast) image of the cells. (e–g) Co-transfection of mCherry-Apobec3 and GFP-DND1 expression constructs into 293T cells. Cells were fixed with 2% paraformaldehyde before visualization of fluorescent signal by confocal microscopy. (e) Image of red fluorescence due to mCherry-APOBEC3 (red filter; excitation at 548 nm); (g) green fluorescence due to GFP-DND1 (green filter, excitation at 488 nm) and (f) merged image of red and green filters showing APOBEC3 and DND1 co-localize to cytoplasm in 293T cells.

In contrast to results in COS7 cells, although both GFP-DND1 and mCherry-APOBEC3 were expressed in the cell cytoplasm, the sequestration of GFP-DND1 and mCherry-APOBEC3 to perinuclear regions was somewhat less obvious in NIH3T3 cells (Fig. 6a, b and d) and less so in 293T cells (Fig. 6 e, g and f). One explanation as to why we observed co-localization and sequestration in COS7 but not in 293T cells could be because COS7 cells may harbor cellular factors that facilitate sequestration of GFP-DND1 and mCherry-APOBEC3. The confocal microscopy results of DND1 and APOBEC3 localization were identical when GFP or mCherry were cloned either as N-terminus or C-terminal fusion proteins to DND1 or APOBEC3, respectively (data not shown).

Human GFP-APOBEC3 orthologs (APOBEC3D, APOBEC3F and APOBEC3G) have been reported to be expressed in the cytoplasm [38], [33] similar to the distribution that we observe for mouse APOBEC3. More detailed studies show that human APOBEC3 localizes to cytoplasmic bodies P-bodies and stress granules [29], [31]. Future work will examine if both DND1 and APOBEC3 also localize to P-bodies in cells.

In our transfection experiments using fluorescently-tagged APOBEC3, we did not observe localization of mCherry-APOBEC3 to discreet cytoplasmic bodies but to be generally present in the cytoplasm (Fig. 5d). Thus, our results are somewhat different from reports of APOBEC3 localization to discreet cytoplasmic bodies [29]. This difference in observations could be due to variability in cell lines, transfection conditions or because mouse APOBEC3 was used in our studies as opposed to human APOBEC3G in the other studies.

## Discussion

### APOBEC3 interacts with DND1

We have used several experimental methods to demonstrate that DND1 interacts with APOBEC3 *in vitro* as well as *in vivo* in mammalian cells and tissues. First, APOBEC3, generated *in vitro,* interacts specifically with DND1. We ruled out the presence of contaminating RNA or DNA in the *in vitro* translation mix that facilitates DND1-APOBEC3 interaction. We observed that mouse and human APOBEC1 were also able to bind to DND1 but there was significant non-specific binding of APOBEC1 to the Sepharose 4B beads as well. This suggests that APOBEC1 may also interact with DND1, although the interaction may be weaker and with lower affinity.

Second, DND1 pulled-down APOBEC3 from mammalian cells when tagged proteins were generated in transfected cells. Thus, DND1 and APOBEC3 are found in the same protein complex in mammalian cells. Recent reports indicate that human APOBEC3G interacts with a number of cellular proteins with RNA binding domains [29], [31]. In most cases, direct interaction between APOBEC3G and the proteins did not appear to be the case and the interaction between the proteins was likely through some shared mRNA. In other cases, for example, interaction of APOBEC3 with the proteins, Argonaute-1 and Argonaute-2, appeared not to be facilitated by RNA because the interaction was RNAse-resistant [29]. In the same study, 293T cells transfected with tagged-APOBEC3G resulted in the ‘pull-down’ and identification of a number of other cellular protein(s) [29] many of which were RNA binding proteins. Among the other proteins that were ‘pulled-down’ together with APOBEC3G was the HuR protein. HuR is an AU-rich element binding protein which has been shown to bind to the 3′-UTR of CAT-1 mRNA to prohibit access to miR-122 [39]. Thus, HuR is another RNA binding protein with a functional role similar to DND1.

Third, our studies demonstrate that GST-DND1 pulls-down endogenous APOBEC3 from mouse testes. In this case, pull-down of the endogenous, mouse testes APOBEC3 was more effective by DND1α compared to DND1β. This was unlike the interaction of *in vitro* translated APOBEC3 with GST-DND1α and GST-DND1β, where both isoforms were equally able to interact with APOBEC3. One explanation for this is that testes APOBEC3 may be post-translationally modified such that it influences its interaction with DND1α or DND1β.

Fourth, fluorescent-protein tagged DND1 and APOBEC3 proteins sequestered near peri-nuclear sites in COS7 cells. This phenomenon appears to be cell specific and was observed in COS7 cells but not in some other cell types. An explanation for this may be that additional cellular factors present in COS7 are responsible for mediating sequestration of DND1 and APOBEC3. The pull-down experiments of DND1 with APOBEC3 do not unambiguously indicate a direct interaction of the two proteins. However, taking into consideration the cell-type specific sequestration of fluorescently tagged DND1 and APOBEC3, it suggests that the interaction of DND1 and APOBEC3 may likely be mediated by other factors in the cell.

Studies by three independent groups report the localization of human APOBEC3G to P-bodies and stress granules in 293T cells [29]–[31]. The evidence suggests that the cytidine deaminase activity of APOBEC3G is likely inhibited in these cytoplasmic granules [40], [28], [30]. In light of this, the consequence of DND1-APOBEC3 interaction for either APOBEC3 or DND1 function remains to be elucidated.

In addition to the experimental demonstration of DND1-APOBEC3 interaction, we found that both *Dnd1* and *Apobec3* transcripts are detected in germ cells and in the developing embryonic gonads. The transcripts are present in germ cells and in genital ridges during embryonic stages when DND1 function is required for germ cell viability. Moreover, lack of *Dnd1* at these embryonic stages also results in initiation of germ cell tumors in the 129 strain male [5], [7].

### Potential function of DND1-APOBEC3 interaction

One function of APOBEC3 is that it is an antiretroviral factor and inactivates exogenous and endogenous retroviruses [20]. Human APOBEC3 (APOBEC3G) suppresses a variety of retroviruses including Vif (virion infectivity factor)-deficient human immunodeficiency virus type 1 (HIV-1) [41]–[44], [38], [45]–[52]. APOBEC3 also restricts transposition of endogenous retrotransposons such as MusD, intracisternal A-particle (IAP) [53], [20] and long interspersed nuclear elements, LINE-1 (L1) [54]–[56], [33]. Although cytidine deamination appears to be the primary mechanism by which APOBEC3 inhibits retrovirus replication [43], [38], [46], [50], there is also a large body of evidence suggesting some novel, yet undefined, deaminase independent mechanism for the antiviral function of APOBEC3 [54]–[56], [33]. Mouse APOBEC3 has also been shown to have anti-retroviral activity [20]. Mouse APOBEC3 has two cytidine deaminase domains (CDD) [57], [19]. The proximal CDD is involved in deamination whereas the distal CDD is involved in dimerization of APOBEC3 proteins and for viral encapsidation [57].

Recent reports indicate additional cellular functions of APOBEC3. APOBEC3 is able to inhibit miRNA-mediated repression of mRNA. APOBEC3 appears to allow mRNA to move from P-granules to polysomes [28].

The role of APOBEC3 in suppressing miRNA mediated mRNA repression parallels recent reports on the role of DND1 in suppressing miRNA-mediated mRNA repression [8]. It is not clear if DND1 sequesters mRNA to prevent its access to miRNA or whether DND1 physically displaces miRNA RISC complex from mRNA [8], [12]. In this respect, it will be important to examine if DND1 and APOBEC3 function synergistically or antagonistically to modulate miRNA function.

The inactivating mutation in the *Dnd1* gene in *Ter* mice causes either sterility or testicular germ cell tumor development. We report here that DND1 interacts with APOBEC3. The functional consequences of DND1 interaction with APOBEC3 remains to be established. One possibility is that DND1 may modulate some specific antiviral or anti-retrotransposion activity of APOBEC3. DND1 may be a cellular factor that is necessary for regulating some aspect of APOBEC3 activity, especially in germ cells.

Another possibility is that DND1 and APOBEC3 function synergistically or antagonistically to modulate miRNA mediated repression of mRNA. The 3′-UTR of mRNAs generally contains multiple miRNA binding sites as well as binding sites for different RNA binding proteins. In light of the reports that both DND1 and APOBEC3 bind to mRNA to inhibit miRNA mediated repression, and taking into consideration our observation of DND1-APOBEC3 interaction, our studies implicate a possible role of DND1-APOBEC3 interaction in modulating miRNA-mediated mRNA repression. Understanding the role of DND1-APOBEC3 interaction will not only shed light on the development of testicular germ cell tumors in mice and humans but may also have profound implications for our understanding of the mechanism of how cancers in general originate in humans.

## Materials and Methods

### Cell Lines

NIH3T3, COS7 and human embryonic kidney (HEK) 293T cells were from ATCC.

### RT-PCR of APOBEC transcripts

RT-PCR was performed as described [32] to amplify *Apobec*-1, *Apobec*-2 and *Apobec*-3 cDNA from 129 testes mRNA. Primers flanking the start and stop codons of each gene were used for the amplifications and were as follows:

Apo1-F: 5′-cagagcaagatgagttccgagac-3′ and
Apo1-R: 5′-caactcccagaagtcatttc-3′ for Apobec-1;
Apo2-F : 5′-cacagttcctccatggctcaga-3′ and
Apo2-R: 5′-cgagctctgttgcctacttcag-3′ for *Apobec*-2;
Apo3-F: 5′-cagagctgggatgggaccattctg-3′ and
Apo3-R: 5′-gaatctcttcttgcctctcaagac-3′ for *Apobec*-3;
Aicd-F 5′-gaccgatatggacagccttctg-3′ and
Aicd-R 5′-gctttcaaaatcccaacatac-3′ for AICD.

### In vitro transcription and translation

The full-length cDNA of the *Apobec*s were cloned into pBK-CMV, sequence verified and used as templates in *in vitro* transcription/translation reactions. TnT Coupled Reticulocyte Lysate Systems (Promega) was used to generate [^35^S]methionine-labeled APOBEC proteins in *in vitro* transcription/translation reactions. The labeled proteins were analyzed on NuPAGE-Mes gels (Invitrogen). 25% of the translated proteins were loaded for analysis in each lane of the gel. The transcription/translation reactions of equal amounts the *Apobec1*, *Apobec2* and *Apobec3* templates generated [^35^S]methionine-labeled proteins of the expected sizes, as determined by electrophoreses. The calculated molecular weight for APOBEC1 is 25.5 KDa, 25.7 KDa for APOBEC2, 47.4 KDa for APOBEC3 protein containing 8 exons and 51 KDa for APOBEC3 protein containing 9 exons. For controls, human APOBEC1 (hA1) (calculated molecular weight, 22.8 KDa) and human ACF (65 KDa) were also generated by *in vitro* transcription/translation of cloned expression constructs.

### Generation and purification of GST-DND1

The cDNA of the two isoforms of mouse DND1 (isoforms α and β) were cloned in-frame into pGEX-2TK (amersham pharmacia biotech) [32]. The GST(glutathione S-transferase)-DND1 fusion proteins were induced by IPTG (isopropyl-β-D-thiogalactoside), extracted from bacteria by sonication and affinity purified using Glutathione Sepharose 4B beads. GST proteins were also generated in a similar manner for use in control reactions.

### In vitro interaction reactions

Equal amounts of *Apobec* cDNA cloned into pBK-CMV (1 mg) were used as templates in *in vitro* transcription/translation reactions (TnT Coupled Reticulocyte Lysate Systems, Promega) to generate [^35^S]methionine-labeled APOBEC proteins in a total volume of 100 μL. The amount of each [^35^S]methionine-labeled APOBEC protein generated in the reaction was determined by electrophoreses of 5 μL on a gel.

Equal amounts (40 μL) of each of the [^35^S]methionine-labeled APOBEC proteins were placed into two tubes. One tube of the [^35^S]methionine-labeled APOBEC proteins was incubated with purified GST-DND1 and Glutathione (GST) Sepharose 4B beads. The second tube (control) was incubated with beads only. Glutathione Sepharose 4B binds to GST and GST-fusion proteins (in this case, GST-DND1) and should therefore “pull-down” proteins that associate with GST-DND1. At the end of the incubation period, the tubes were centrifuged to pellet the Sepharose 4B beads. The beads were washed to reduce non-specific binding. Loading dye was added to the beads before heating to 95^0^C to release the proteins from the beads into the loading dye. Equal volumes of the loading dye were electrophoresed on NuPAGE-Mes gels (Invitrogen) to assess the binding of the radiolabelled APOBEC proteins to GST-DND1.

To test whether the *in vitro* binding of GST-DND1 to APOBEC3 is dependent on RNA or DNA that may be present in the *in vitro* transcription/translation reactions, [^35^S]labeled APOBEC-3-GST-DND1 complexes were treated for 1 h with 0.08 U RNase (RNAse, DNase-free from bovine pancreas, Roche) or 5 U DNase (DNAse 1, RNase-free from bovine pancreas).

### Pull-down of APOBEC3 with DND1 in mammalian cells

*Dnd1* and *Apobec* cDNAs were cloned in frame into mammalian expression vectors (pCMV-HA or pCMV-Myc, Clontech) to generate HA(hemagglutinin) epitope-tagged DND1 or myc-tagged APOBEC proteins. Both HA-*Dnd1* and myc-*Apobec* plasmid constructs were co-transfected into human embryonic kidney (HEK) 293T cells. The transfected 293T cells were lysed after 48 h, and immunoprecipitation using anti-HA antibody (abm) was performed to “pull-down” HA-DND1 and associated proteins. After electrophoresis and transfer to membranes, western blotting was performed using anti-myc antibody (Invitrogen). Control aliquots of cell lysates were not incubated with antibody.

### RNA isolation from EG cells and genital ridges

EG cells were isolated from hybrid strains of B6C3 F1 mice [35]. The cells were grown on mitomycin C-treated mouse embryo fibroblasts. While in culture, some EG cell plates were periodically stained with alkaline phosphatase chromogen (abcam) to ensure they had not differentiated.

RNA isolation from male genital ridges from 129 mouse embryos at E13.5 stages was as described [32].

### Fluorescent protein tagged-APOBEC3 and DND1

Mouse *Dnd1* was cloned in frame and fused to fluorescent GFP (green fluorescent protein) either in the N or C-terminus in pEGFp-N3 (Clontech) and pEGFP-C1 (Clontech) vectors. Mouse Apobec3 (9-exon isoform) was cloned in frame and fused to monomeric cherry fluorescent protein (mCherry) in the pRSET-B mCherry vector [58]. The cherry fluorescent protein was substituted into the ECFP sites in the pECFP-C1 (Clontech) and pECFP-N1 (Clontech) vectors so the multiple cloning sites of C1 and N1 could be used to clone in and generate C and N-terminal cherry fusion products.

The GFP-*Dnd1* and mCherry-*Apobec*-3 were transfected separately into 293T or COS7 cells. After 20 hours, transfected cells were fixed with 2% paraformaldehyde before visualization of fluorescent signal by *Zeiss* LSM 510 Confocal Microscope. Green fluorescence due to GFP-DND1 was imaged using green filter (excitation at 488 nm); red fluorescence due to mCherry-APOBEC-3 was imaged using red filter (excitation at 548 nm).
